# Therapeutic potential of natural products from Traditional Chinese Medicine in the treatment of osteoporosis

**DOI:** 10.3389/fphar.2026.1779967

**Published:** 2026-03-31

**Authors:** Weiting Xiao, Wei Liu, Xionglong Wang, Qiongli Zeng, Yang Tang, Mingjun Xia, Linli Dong, Shujun Fei, Wei Yue, Shunxiang Li, Wen Ouyang

**Affiliations:** 1 School of Pharmacy, Hunan University of Chinese Medicine, Changsha, China; 2 Key Laboratory of Modern Research of TCM, Education Department of Hunan Province, Changsha, China; 3 Analysis of Complex Effects of Proprietary Chinese Medicine, Hunan Provincial Key Laboratory, Yongzhou, China; 4 Liuyang Jinyang Hospital, Liuyang, China

**Keywords:** flavonoids, natural products, osteoporosis, saponins, Traditional Chinese Medicine

## Abstract

Osteoporosis (OP) is a systemic bone disorder characterized by reduced bone mass and deterioration of bone microarchitecture. Despite the widespread use of classic medicines such as parathyroid hormone and bisphosphonate in clinical practice, their adverse effects continue to be a major concern. Consequently, the exploration of safe and effective therapeutic agents derived from botanical drugs has emerged as a key research focus. Traditional Chinese medicine (TCM) constitutes a valuable therapeutic modality for osteoporosis, attributed to its abundant botanical resources, multi-target modulatory effects, and favorable safety and effectiveness validated by long-term clinical practice. In recent years, natural products from TCM have gained increasing attention for their potential in the prevention and treatment of osteoporosis. This review provides a comprehensive summary of the diverse classes of metabolites currently investigated for OP management, along with their underlying mechanisms of action. By elucidating the pharmacological mechanisms of these bioactive metabolites, this study aims to provide a reference for evaluating their potential as alternative or adjunctive therapeutic strategies in osteoporosis management.

## 1 Introduction

In the process of bone remodeling, the dynamic balance of bone metabolism is mainly maintained by the precise coupling of osteoblast-mediated bone formation and osteoclast-mediated bone resorption (Weivoda and Bradley, 2023). Osteoclasts first mediate bone resorption, and the released regulatory factors recruit and activate osteoblast precursors to differentiate into osteoblasts, thereby initiating new bone formation. Through this continuous cycle of old bone replacement, the bone efficiently repairs micro-damage, thereby maintaining bone strength and mineral homeostasis (Sun et al., 2025; Bolamperti et al., 2022). When bone resorption outpaces bone formation, the disruption of bone metabolism equilibrium can lead to pathological bone loss, which in turn develops into osteoporosis, manifested as thinning of trabecular bone and deterioration of bone microstructure (Zheng et al., 2023; Wang S. et al., 2025). These structural impairments significantly increase fracture risk. According to statistics, fragility fractures become increasingly prevalent after age 55 in women and 65 in men, often resulting in a large number of bone-related complications and increasing mortality (Zhang Y. K. et al., 2025). Current pharmacological interventions for osteoporosis are categorized based on their mechanisms of action, including antiresorptive agents, bone-forming agents, and dual-action drugs (Ramchand and Leder, 2024). Although widely used clinical agents like bisphosphonates, selective estrogen receptor modulators (SERMs), and parathyroid hormone analogs show favorable therapeutic efficacy, prolonged use of these medications can have negative side effects, such as osteonecrosis of the jaw and rebound bone loss after drug withdrawal (Mutlu et al., 2025). These clinical challenges underscore the need for safer and more sustainable therapeutic alternatives.

Natural products occupy a critical position in the prevention and treatment of chronic diseases, representing a valuable reservoir for exploring promising therapeutic candidates. In TCM, osteoporosis is classified as “bone impotence” or “bone paralysis,” with its pathogenesis closely associated with deficiencies in the liver and kidney as well as spleen-stomach weakness (Cao G. et al., 2024). Clinical applications of TCM for osteoporosis management typically focus on botanical drugs with “tonify kidney and strengthen bone” properties (Lin et al., 2023), including *Drynaria roosii* Nakaike (Gusuibu), *Epimedium brevicornu* Maxim. (Yinyanghuo), and *Achyranthes bidentata* Blume (Niuxi) etc. Modern pharmacological investigations have revealed that flavonoids, saponins, and alkaloids isolated from them can significantly inhibit bone resorption or promote bone formation, or exert dual regulatory effects (Su et al., 2025; Yang et al., 2023; Zhuo et al., 2022). Compared to conventional anti-osteoporotic drugs, natural products demonstrate distinct advantages including fewer adverse reactions and multi-target therapeutic potential. Their development addresses limitations of current treatments while offering novel intervention strategies through specific signaling pathways modulation. This review summarizes recent advances in anti-osteoporotic natural products from TCM, with particular emphasis on their molecular mechanisms and therapeutic potential.

For this study, a comprehensive literature search was performed using the PubMed (https://pubmed.ncbi.nlm.nih.gov/) and CNKI (https://www.cnki.net/) databases, with search terms including “natural products,” “traditional Chinese medicine,” “osteoporosis,” “osteoblast,” “osteoclast,” “bone” and related keywords. Literature searches were conducted from database inception to January 2026, with a primary focus on studies published between 2016 and 2025. Inclusion criteria were original research articles and reviews on natural products derived from TCM for treating osteoporosis, studies with well-defined mechanisms of action, and studies with rigorous experimental designs. Exclusion criteria were outdated and repetitive literatures, studies with incomplete information, studies unrelated to osteoporosis, and studies with flawed experimental designs. The extracted data included metabolites name, source species, experimental model, and mechanism of action.

## 2 Common therapeutic targets for osteoporosis

### 2.1 Bone formation

#### 2.1.1 Wnt/β-catenin pathway

The Wnt/β-catenin signaling pathway is a core pathway regulating bone formation. Dysregulation of this signaling pathway may result in inhibition of osteoblast differentiation, reduced bone formation, and bone loss. Sclerostin (SOST) is a classical inhibitor of Wnt/β-catenin signaling, which inhibits the binding of Wnt to low-density lipoprotein receptor-related protein 5/6 (LRP5/6) and frizzled, blocking pathway activation, and suppresses bone formation (Zhao et al., 2023; Hu et al., 2024). Clinically, romosozumab is a monoclonal antibody targeting SOST, which relieves LRP5/6 inhibition by neutralizing SOST and restores Wnt/β-catenin pathway activity, thereby promoting osteogenic differentiation and inhibiting bone resorption (Wu et al., 2023).

#### 2.1.2 BMP/SMAD/RUNX2 pathway

Bone morphogenetic protein (BMP) was originally discovered based on its ability to induce bone formation. It belongs to the transforming growth factor-β (TGF-β) superfamily. BMP2 is widely used in bone research because of its significant osteoinductive activity (Lee et al., 2025). Studies have shown that BMP2-deficient mice exhibit obvious bone development abnormalities, including reduced bone volume, decreased bone strength, sparse trabecular structure, and increased cortical bone porosity (Toth et al., 2021). The Smad pathway is a classical pathway mediated by BMP2. When BMP2 binds to its cell surface receptors, it triggers a downstream signaling cascade, leading to phosphorylation of Smad1/5/8. Subsequently, phosphorylated Smad1/5/8 forms a complex with Smad4 and translocates into the nucleus, thereby regulating the expression of osteoblast-specific transcription factors, including runt-related transcription factor 2 (Runx2) and Osterix (Osx), as well as osteogenic markers such as alkaline phosphatase (ALP) and osteocalcin (OCN), ultimately promoting the osteogenic differentiation of mesenchymal stem cells (MSCs) (Du et al., 2025; Yao et al., 2022). The expression level of Runx2 is closely related to BMP2. Research has found that in chondrocyte-specific BMP2 knockout mice, Runx2 expression is significantly downregulated, indicating that BMP2 signaling positively regulates Runx2 expression (Ding et al., 2023).

### 2.2 Bone resorption

#### 2.2.1 RANKL/RANK/OPG pathway

The RANKL/RANK/OPG signaling pathway plays a pivotal role in the pathogenesis of osteoporosis, and its dysregulation is closely associated with excessive osteoclast activation. During bone remodeling, osteoblast-derived receptor activator of nuclear factor-κB ligand (RANKL) binds to receptor activator of nuclear factor-κB (RANK) on osteoclast precursors, promoting osteoclast precursor differentiation via pathways such as NF-κB and MAPK, and also prolonging the survival of mature osteoclasts. Osteoprotegerin (OPG) secreted by osteoblasts can bind to RANKL to inhibit RANKL-RANK interaction, reduce osteoclast formation and maintain the dynamic balance between bone resorption and bone formation (Wang K. et al, 2024; Yin et al., 2025). When RANKL expression is upregulated or OPG secretion is reduced, the RANKL-RANK signaling pathway is abnormally activated, resulting in enhanced bone resorption. Denosumab, a clinically used RANKL inhibitor, competitively binds to RANKL, thereby suppressing RANK/RANKL/OPG pathway activation and inhibiting osteoclast-mediated bone resorption. However, rapid bone loss may occur following treatment discontinuation (Lu J. et al, 2023).

#### 2.2.2 M-CSF/c-Fms system

**FIGURE 1:**
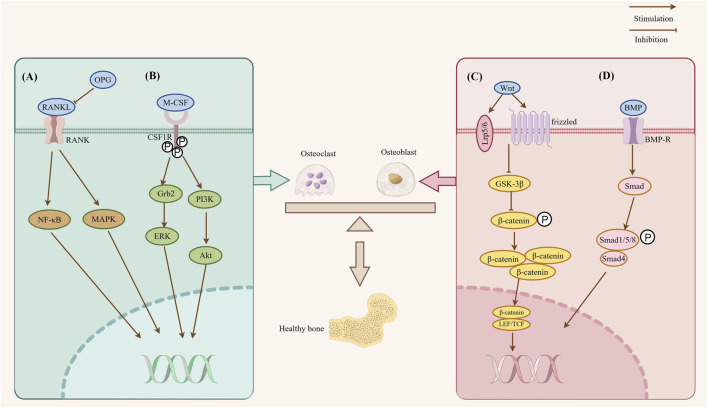
The diagram illustrates the key signaling pathways involved in bone remodeling. **(A)** RANKL/RANK/OPG pathway: RANKL binding to its receptor RANK induces the activation of NF-κB and MAPK pathway, thereby promoting osteoclast formation; OPG blocks this effect by competitively binding to RANKL. **(B)** M-CSF/c-Fms system: M-CSF binding to its receptor CSF1R triggers phosphorylation of specific tyrosine residues in the receptor cytoplasmic tail, which in turn induces recruitment of Grb2 and PI3K and activates downstream signaling pathways such as Akt and ERK to promote osteoclast formation. **(C)** Wnt/β-catenin pathway: Upon binding to the Frizzled receptor and LRP co-receptor on the cell membrane, Wnt ligand inhibits the phosphorylation of β-catenin by GSK-3β, leading to the accumulation of non-phosphorylated β-catenin in the cytoplasm. Subsequently, β-catenin translocates into the nucleus, where it associates with TCF/LEF transcription factors to activate downstream gene expression, thereby promoting osteoblast differentiation. **(D)** BMP/SMAD/RUNX2 pathway: BMP ligands bind to BMP receptors, leading to the activation and C-terminal phosphorylation of Smad1/5/8. The phosphorylated Smad1/5/8 then forms a complex with Smad4, which translocates into the nucleus to regulate the transcription of osteogenic genes, thereby promoting osteoblast differentiation (This schematic diagram was drawn by using Figdraw).

Macrophage colony-stimulating factor (M-CSF) is essential for the entire process from the production of osteoclast precursors to the formation and survival of mature osteoclasts. Upon M-CSF binding to its receptor c-Fms (CSF1R), the receptor is phosphorylated at a specific tyrosine residue in its cytoplasmic tail. These phosphorylated tyrosine residues on the receptor recruit signaling molecules such as growth factor receptor-bound protein 2 (Grb2) and phosphatidylinositol 3-kinase (PI3K). This process triggers the activation of the extracellular signal-regulated kinase (ERK) and protein kinase B (Akt) pathways, which promote the survival and proliferation of osteoclast precursors (Mun et al., 2020; Chen H. et al, 2024). Additionally, M-CSF cooperates with β3 integrin to regulate actin remodeling in osteoclasts, allowing osteoclasts to spread, migrate, fuse and form actin rings to promote bone resorption (Inoue et al., 2023). Studies demonstrate that genetic deficiencies in M-CSF (op/op mice) or c-Fms mutations (Csf1r mutations) impaired osteoclastogenesis, resulting in severe osteopetrosis in murine models (Hume et al., 2022; Zhu M. et al, 2021). The M-CSF/c-Fms pathway exhibits tight functional synergy with RANKL/RANK signaling. Mechanistically, M-CSF not only enhances sensitivity of osteoclast precursor cells to RANKL but also upregulates RANK expression, thereby potentiating osteoclast differentiation (Figure 1) (Riedlova et al., 2023).

### 2.3 Androgen and estrogen

Androgen and estrogen deficiency represents a primary etiological factor for osteoporosis in postmenopausal women and aging men (Luo et al., 2021). Estrogen deficiency triggers upregulation of proinflammatory cytokines such as IL-6 and TNF-α, which accelerate osteoclast differentiation. Concurrently, it disrupts the RANKL/OPG equilibrium, ultimately enhancing bone resorption (Cheng et al., 2022). Furthermore, estrogen deficiency also affects the function of osteoblasts, which is manifested by a decrease in the number of cells and a shortened lifespan, resulting in bone formation disorders (Schiavi et al., 2021). Clinical studies reveal an age-associated decline in serum testosterone levels in males. Testosterone enhances osteoblast differentiation *via* androgen receptor (AR)-mediated upregulation of tenascin-C (TNC), while testosterone deficiency leads to relative increases in osteoclast activity (Xie et al., 2025). In addition, aging reduces aromatase activity, diminishing the conversion of testosterone to estrogen and consequently impairing estrogen receptor (ER)-mediated bone protective effects (Tenuta et al., 2025). Therapeutically, SERMs such as raloxifene demonstrate tissue-specific ER agonism in bone, effectively suppressing osteoclast activity and increasing bone mineral density (BMD) (Motlani et al., 2023).

## 3 Traditional Chinese medicine for osteoporosis

According to TCM, the primary pathogenesis of osteoporosis involves deficiency of the zang-fu organs, among which kidney deficiency is the main cause, liver deficiency is a key factor, and spleen deficiency is also a significant contributor. Blood stasis serves as the pathological basis of osteoporosis. These TCM pathogenic theories have guided the OP treatment, with therapeutic objectives centered on nourishing the kidney and liver, invigorating spleen qi, and activating blood circulation to resolving blood stasis (Lin et al., 2023). With the development of modern pharmacological approaches, these classical TCMs have been systematically investigated to elucidate their material basis and mechanisms of action (Tables 1–3). Owing to its multi-component and multi-target characteristics, TCMs exert holistic regulatory effects through multiple pathways and biological processes, demonstrating unique advantages in the treatment of OP and holding potential for development as novel anti-osteoporotic agents.

**TABLE 1:** Summary of *in vivo* studies on representative anti-osteoporotic botanical drugs.

Source species	Traditional use	Group of metabolites	Metabolite/type of extract assessed	Bioactive metabolites	Animal models	Range of dosage	Mechanism/effect	Bibliography
*Epimedium brevicornu* Maxim	Nourish the liver and kidneys, strengthen the muscles and bones, alleviate rheumatic conditions	Flavonoid	Purified metabolite	Icariin	OVX rat	125, 250, 500 mg/kg/day for 3 months	Regulates the Cullin 3/Nrf2/OH signaling pathway to suppressing osteoclastogenesis	Si et al. (2024)
			Purified metabolite	Epimedin A	OVX rat	5, 10, 20 mg/kg/day for 90 days	Inhibits TRAF6/PI3K/Akt/NF-κB pathway	Li J.et al. (2024)
			Purified metabolite	Epimedin B	DOP rat	10, 20, 40 mg/kg/day for 8 weeks	Reduces blood glucose in rats, promotes bone trabecula formation, improves bone microstructure, reduces the number of bone marrow adipocytes, regulates OPG/RANKL axis and inhibits inflammation	Zhang et al. (2025b)
			Purified metabolite	Icariside II (Baohuoside I)	OVX rat	100 mg/kg/day for 18 weeks	Reduces bone marrow fat content, inhibits adipogenic differentiation of BMSCs, and suppresses S100A16 expression	Li D. et al. (2024)
*Drynaria roosii* Nakaike	Tonify the kidneys and strengthen the bones, activate blood circulation, and dispels wind	Flavonoid	Purified metabolite	Naringin	OVX mice	10, 20, 40 mg/kg/day for 8 weeks	Targets the TLR2/MyD88/NF-κB pathway	Li Z. et al. (2025)
			Purified metabolite	Naringenin	simulated microgravity (SMG) mice	60, 100 mg/kg/day for 4 weeks	Activates the Nrf2/HO-1 pathway	Cao S. et al. (2024)
*Cullen corylifolium* (L.) Medik	Tonify kidney and strengthen bone, secure essence and reduce urination	Flavonoid	Purified metabolite	Neobavaisoflavone	OVX mice	30 mg/kg/day for 6 weeks	Inhibits osteoclastogenesis and promots osteogenesis	Chen et al. (2020)
			Purified metabolite	Corylin	OVX mice	10 mg/kg, twice weekly for 6 weeks	Inhibitis the NF-κB pathway to suppress osteoclast differentiation	Li X. Q. et al. (2021)
		Coumarin	Purified metabolite	Isopsoralen	GIOP mice	5, 10, 20 mg/kg/day for 4 weeks	Regulates cGMP/PKG pathway-mediated purine metabolism and promotes osteoblast differentiation	Liu D. et al. (2024)
			Purified metabolite	Psoralen	OVX mice	10, 20, 40 mg/kg/day for 28 days	Inhibits HSD17B2 activity, prevent the inactivation of E2, increases levels of endogenous estrogen, improves bone metabolism-related indices	Lu Y. et al. (2023)
*Eucommia ulmoides* Oliv	Tonify liver and kidney, strengthen bones and muscles	Lignan	Purified metabolite	Pinoresinol diglucoside	OVX mice	5, 10 mg/kg/day for 8 weeks	Regulates the microbiota-gut-bone axis; Promotes osteogenic differentiation of BMSCs and increases bone mineral density	Li N. et al. (2025)
					SMG rat	18, 36, 72 mg/kg/day for 21 days	Promotes the osteogenic differentiation of BMSCs, inhibits the apoptosis of bone cells, reduces oxidative stress and inflammatory response, and regulates the ratio of RANKL/OPG to inhibit bone resorption	Xuan et al. (2026)
*Dipsacus asper* Wall. ex DC.	Tonify liver and kidney, strengthen bones and muscles, arrest metrorrhagia and metrostaxis	Saponin	Purified metabolite	Asperosaponin VI	DOP mice	50 mg/kg, three times per week for 6 weeks	Inhibits DNMT and Restores GPX4 expression to reduce osteoblast ferroptosis	Wei F. et al. (2024)
		Polysaccharide	Water extraction	DAP	OVX rat	50, 200 mg/kg/day for 12 weeks	Upregulates the expression of VEGF and OPG, downregulates the expression of RANK and RANKL, and activates the PI3K/Akt/eNOS signaling pathway	Sun et al. (2019)
*Gynochthodes officinalis* (F.C.How) Razafim. and B.Bremer	Tonify kidney yang, strengthen bones, dispel wind and eliminate dampness	Polysaccharide	—	MOP	OVX rat	400 mg/kg/day for 4 weeks	Increases the expression of miR-21 in rBMSCs to enhances rBMSC osteogenic differentiation and inhibits adipogenic differentiation	Wu et al. (2022)
			Water extraction	MOP-D2N1	GIOP mice	100, 300 mg/kg/day for 9 weeks	Regulates oxidative stress pathway to promote osteogenic activity and restores bone quality	Lai et al. (2025)
*Cistanche deserticola* Ma	Tonify kidney yang, benefit essence and blood, moisten dryness and promote bowel movements	Polysaccharide	Water extraction	*Cistanche deserticola* Polysaccharides	SAMP6 mice	400 mg/kg/day for 12 weeks	Activates the Wnt/β-catenin signaling pathway, downregulates the expression of RANKL and p-β-catenin, and upregulates the expression of BMP-2, OCN, OPG and p-GSK-3β	Wang F. et al. (2021)
​	​	Phenylethanoid glycoside	Purified metabolite isolated from its 70% ethanol extraction	Acteoside	OVX mice	20, 40, 80 mg/kg/day for 12 weeks	Downregulates the RANKL/RANK/TRAF6 axis, subsequently suppresses NF-κB, and stimulates PI3K/Akt signaling	Yang et al. (2019)
					GIOP rat	50, 100 mg/kg/day for 8 weeks	Activats the PI3K/Akt/mTOR pathway	Li S. et al. (2023)
			Purified metabolite isolated from its 70% ethanol extraction	2′-acetylacteoside	OVX mice	10, 20, 40 mg/kg/day for 12 weeks	Through RANKL/RANK/TRAF6-mediated NF-κB/NFATc1 pathway, downregulates expression of RANK, TRAF6, IκB kinase β, NF-κB and NFATc1	Li Y. et al. (2020)
			Purified metabolite isolated from its 70% ethanol extraction	6- acetylacteoside	OVX mice	10, 20, 40 mg/kg/day for 12 weeks	Prevents bone resorption by regulating the RANKL/RANK/OPG mediated NF-κB and PI3K/Akt pathways	Liu et al. (2022)
*Cnidium monnieri* (L.) Cusson	Warm and tonify kidney yang, dispel wind, eliminate dampness and relieve itching	Coumarin	Purified metabolite	Osthole	12-month-old mice	5 mg/kg/day for 4 weeks	Attenuates osteoclast formation by stimulating the activation of β-catenin-OPG signaling	Jin et al. (2021)
*Rehmannia glutinosa* (Gaertn.) Libosch. ex DC.	Nourish yin and tonify blood	Iridoid	Purified metabolite	Catalpol	OVX rat	5, 10, 20 mg/kg/day for 12 weeks	Promots osteoclast apoptosis via the Sirt6-ERα-FasL axis	Chen S. et al. (2024)
					GIOP mice	20, 100 mg/kg/day for 6 weeks	Upregulates PKD1 protein expression, reduces oxidative stress, and promotes expression of bone formation-associated markers	Xu et al. (2023)
*Lycium barbarum* L	Nourish the liver and kidneys, tonify the essence, and brighten the eyes	Polysaccharide	Water extraction	*Lycium barbarum* polysaccharides	Healthy WT adult C57BL/6J mice (male, 2 months old) 14-month-old naturally aging male C57BL/6J mice and adult C57BL/6J female mice (2 months old)	40 mg/kg/day for 2 months	Increases bone mass and bone strength, and promotes osteoblast proliferation, differentiation	Sun et al. (2023)
​	​	​	Water extraction (followed by DEAE Sepharose™ Fast Flow column and Sephacryl S-300 HR column)	LBP1C-2	Healthy WT adult C57BL/6J mice (male, 2 months old) 14-month-old naturally aging male C57BL/6J mice and adult C57BL/6J female mice (2 months old)	40 mg/kg/day for 2 months	Increases bone mass and bone strength, and promotes osteoblast proliferation, differentiation, and ossification by targeting BMPRIA/BMPRII/Noggin	​
*Cuscuta chinensis* Lam	Tonify kidney, secure essence and reduce urination, brighten the eyes, calm the fetus	Flavonoid	Purified metabolite	Hyperoside	OVX mice	20, 40, 80 mg/kg/day for 10 weeks	Inhibits osteoclast differentiation and protects OVX-induced osteoporosis through the ERα/ITGβ3 signaling pathway	Wei Q. et al. (2024)
					OVX mice	10, 20, 40 mg/kg/day for 6 weeks	Relieves bone resorption by targeting the miR-19a-5p/IL-17A axis	An et al. (2023)
		Polysaccharide	Water extraction	*Cuscuta chinensis* Polysaccharides	OVX rat	400 mg/kg/day for 12 weeks	Restores the balance between osteoblast-regulated bone formation and osteoclast-regulated bone resorption	Liu et al. (2020a)
*Achyranthes bidentata* Blume	Activate blood circulation and unblock collaterals, tonify the liver and kidney, strengthen the muscles and bones, promote urination and relieve strangury	Polysaccharide	Water extraction	ABPB	OVX rat	400 mg/kg/day for 13 weeks	Improves BMD, BMC and bone biomechanical properties, and reduces bone turnover rate	Zhang et al. (2018)
			Water extraction	AB-50	OVX rat	400 mg/kg/day for 13 weeks		Yan et al. (2019a)
			Water extraction	AB-70	OVX rat	400 mg/kg/day for 13 weeks		Zhang et al. (2019)
			Water extraction	AB-90	OVX rat	400 mg/kg/day for 13 weeks		Wang et al. (2017)

**TABLE 2:** Summary of *in vitro* studies on representative anti-osteoporotic botanical drugs.

Source species	Group of metabolites	Metabolite/type of extract assessed	Bioactive metabolites	Cells	Range of dosage	Mechanism	Bibliography
*Epimedium brevicornu* Maxim	Flavonoid	Purified metabolite	Icariin	RAW 264.7	2.5, 5, 10 μmol/L	Regulates the Cullin 3/Nrf2/OH signaling pathway, downregulates ROS levels and suppressing osteoclastogenesis	Si et al. (2024)
				Adipose-derived stem cell (ADSC)	1, 10, 50, 100 μmol/L	Inhibits the Hippo pathway, reduces YAP and TAZ phosphorylation and enhances their transcriptional activity, while simultaneously suppressing PPARγ to promot the osteogenic differentiation of ADSCs and inhibit adipogenic differentiation	Lin et al. (2025)
		Purified metabolite	Epimedin A	RAW 264.7	0.1, 0.2, 0.4 μmol/L	Inhibits the TRAF6/PI3K/Akt/NF-κB pathway to inhibit osteoclast differentiation	Li J. et al. (2024)
		Purified metabolite	Epimedin C	MC3T3-E1	10, 20 μmol/L	Activates the PI3K/Akt signaling pathways to enhance protein expression of Osx, Runx2 and ALPL	Xu et al. (2022)
		Purified metabolite	Icaritin	Bone marrow-derived macrophages (BMMs)	0.01, 0.1, 1 μmol/L	Downregulates the expression of transcription factors NFATc1 and c-fos, inhibits the expression of osteoclast-specific genes RANK, Cathepsin K, MMP9 and TRAP	Huang et al. (2023)
		Purified metabolite	Icariside II (Baohuoside I)	BMSCs	0.1, 1, 10 μmol/L	Increases β-catenin signaling and decreases S100A16 expression to inhibit BMSCs toward adipocyte differentiation	Li D. et al. (2024)
				BMMs	0.01, 0.1, 1 μmol/L	Inhibits osteoclast differentiation by ameliorating the activation of the MAPK and NF-kB pathways and reducing the expression of uPAR	Ma et al. (2022)
	Polysaccharide	Water extraction	EBPC1	Primary osteoblasts	75, 100 μg/mL	Regulates Bax/Bcl2 and accelerates osteogenesis	Lei et al. (2023)
*Drynaria roosii* Nakaike	Flavonoid	Purified metabolite	Naringin	MC3T3-E1	0.1, 0.5, 1 μmol/L	Activates the Wnt/β-catenin pathway to promote osteogenic differentiation	Cui et al. (2025)
				Fibroblasts	50, 100, 150 μmol/L	Activates the Wnt/β-catenin and BMP signaling pathways and regulates OPG/RANKL ratio to inhibit osteoclast differentiation	Yang et al. (2020)
		Purified metabolite	Naringenin	Primary osteoblasts	5, 10 μmol/L	Activates the Nrf2/HO-1 pathway	Cao S. et al. (2024)
				BMSCs	75, 100 μg/L	Regulated SDF-1/CXCR4 signaling pathway to promotes the expression of the SDF-1a gene and protein	Wang Y. et al. (2021)
		Purified metabolite	Neoeriocitrin	hDPSCs	2.5, 5, 10 μmol/L	Inhibits of ubiquitination-mediated degradation and increases autophagy boost osteogenic differentiation	Wu Y. et al. (2025)
*Cullen corylifolium* (L.) Medik	Flavonoid	Purified metabolite	Neobavaisoflavone	MC3T3-E1	16 μmol/L	Upregulates Nrf2/HO-1 signaling pathway to reduce oxidative stress	Zhu Z. et al. (2021)
				RAW 264.7	2, 4, 8 μmol/L	Inhibits the TRAF6 and c-Src to reduce osteoclastogenesis	Chen et al. (2020)
		Purified metabolite	Corylin	RAW 264.7	2,5,10 μmol/L	Inhibits the NF-κB pathway	Li X. Q. et al. (2021)
				Primary osteoblasts	1, 3, 10 μmol/L	Activates the estrogen and Wnt/β-catenin signaling pathway to promote osteogenesis	Yu et al. (2020)
	Coumarin	Purified metabolite	Isopsoralen	OCT1 cells	10, 30, 60 μg/mL	Promotes osteoblast proliferation and differentiation through the BMP2/Runx2/Osx signaling pathway	Zhang et al. (2023)
				BMMCs	10, 20, 30 μmol/L	Inhibits the NF-κB signaling pathway to inhibit RANKL-induced osteoclastogenesis	Zhan et al. (2023)
		Purified metabolite	Psoralen	hBMSCs	0.1, 1, 10 μmol/L	Activates the TGF-β/Smad3 pathway to accelerate osteogenic differentiation of hBMSC	Huang et al. (2021)
*Eucommia ulmoides* Oliv	Iridoid	Purified metabolite	Aucubin	hBMSCs	5, 10, 20 μmol/L	Activates the BMP2/SMADs pathway to promote osteogenic differentiation of hBMSC, inhibit ROS production and oxidative stress	Zheng et al. (2025)
				RAW 264.7	1, 2.5, 5 μmol/L	Inhibits osteoclast differentiation via the Nrf2-mediated antioxidation pathway	Zhang et al. (2021)
	Lignan	Purified metabolite isolated from its MeOH extraction	Pinoresinol diglucoside	MC3T3-E1	10, 30 μmol/L	Regulates BMP2 and β-catenin signals	Park et al. (2020)
		Purified metabolite	Pinoresinol	MC3T3-E1	0.1 μg/L	Induces osteoblast differentiation and mineralization by regulating BMP-2/Runx2 signaling via activation of the cAMP/PKA pathway	Jiang et al. (2019)
*Dipsacus asper* Wall. ex DC.	Saponin	Purified metabolite	Asperosaponin VI	BMSCs	1, 10, 100 μmol/L	Stimulates SMAD, TGF-β1, VEGFA and OPG/RANKL signaling pathways to enhance osteogenesis and inhibit osteoclastogenesis	Chen et al. (2021)
*Gynochthodes officinalis* (F.C.How) Razafim. and B.Bremer	Polysaccharide	—	MOP	BMSCs	10, 50 μg/mL	Enhances rBMSC osteogenic differentiation and inhibites adipogenic differentiation via the miR-21/PTEN/PI3K/Akt axis	Wu et al. (2022)
		Water extraction	MOP70-2	MC3T3-E1	16.1, 32.2, 80.4 μmol/L	Stimulates osteoblastic differentiation by up-regulating osteogenic differentiation-related marker genes	Jiang et al. (2018)
		Water extraction	MOW50-1	MC3T3-E1	5, 10, 20 μg/mL	Promotes osteogenic differentiation of MC3T3-E1 cells by increasing alkaline phosphatase activity	Zhang et al. (2020)
		Water extraction	MOW90-1	MC3T3-E1	50, 100, 250 mg/mL	Upregulates the expression of Runx2, Osx, osteopontin, and osteocalcin	Yan C. et al. (2019)
	Saponin	—	*Morinda officinalis* saponins	HUC-MSCs	5, 25, 100 μg/mL	Promotes osteogenic differentiation of HUC-MSCs by regulating the BMP-SMAD signaling pathway	Zhou J. et al. (2024)
	Anthraquinone	Purified metabolite isolated form its 75% ethanol extraction (CH_2_Cl_2_ fraction)	M13	mMSCs	12.5, 25 μmol/L	Promotes osteogenic differentiation of MSCs via stimulation of the Wnt/β-catenin pathway	Li C. et al. (2023)
​	​	Purified metabolite	Rubiadin-1-methyl ether	BMMs	0.1, 1, 10 mmol/L	Reduces the transcription of BECN1 by inhibiting the activation of NF-κB p65, thereby inhibiting Beclin1-dependent autophagy and RANKL-induced osteoclastogenesis	Cai et al. (2023)
	Iridoid	Purified metabolite	Monotropein	Primary osteoblasts	0.0032, 0.016, 0.08 μmol/L	Attenuates oxidative stress via Akt/mTOR-mediated autophagy	Shi et al. (2020)
*Cistanche deserticola* Ma	Polysaccharide	—	*Cistanche deserticola* Polysaccharides	BMMs	5, 10 μmol/L	Increases the expression of antioxidant enzymes to attenuate RANKL-mediated ROS production in osteoclasts and inhibits nuclear factor of activated T cells and mitogen-activated protein kinase activation	Song D. et al. (2018)
	Phenylethanoid glycoside	Purified metabolite	Acteoside	MC3T3-E1	1 μmol/L	Activates the PI3K/Akt/mTOR pathway	Li S. et al. (2023)
		Purified metabolite	Cistanoside A	Primary osteoblasts	10 μmol/L	Activates autophagy via the Wnt/β-catenin pathway	Chen et al. (2022)
*Cnidium monnieri* (L.) Cusson	Coumarin	Purified metabolite	Osthole	MC3T3-E1	20, 50, 100 μmol/L	Activates the cAMP/CREB signaling pathway to promote osteogenesis	Zhang et al. (2017)
		Purified metabolite	Imperatorin	Saos-2	1, 10 μmol/L	Activates ER pathway to promote osteoblast proliferation and ALP activity, and activate Akt, ERK, and p38 MAPK pathways	Jia et al. (2016)
*Rehmannia glutinosa* (Gaertn.) Libosch. ex DC.	Iridoid	Purified metabolite	Catalpol	RAW 264.7	25, 50, 100 μmol/L	Promotes osteoclast apoptosis via the Sirt6-ERα-FasL axis	Chen S. et al. (2024)
*Lycium barbarum* L	Polysaccharide	—	*Lycium barbarum* polysaccharides	BMSCs	1, 5, 10 μmol/L	Stimulates the autophagy processes and facilitates the formation of autophagosomes and autolysosomes	Wei L. X. et al. (2025)
		Water extraction (followed by DEAE Sepharose™ Fast Flow column and Sephacryl S-300 HR column)	LBP1C-2	BMSCs	4 μmol/L	Targets BMPRIA/BMPRII/Noggin	Sun et al. (2023)
*Cuscuta chinensis* Lam	Flavonoid	Purified metabolite	kaempferol	BMSCs	5, 10 μmol/L	Promotes mitophagy through the Sp1/FUNDC1 signaling pathway and alleviates BMSCs senescence	Wang L. et al. (2025)
				RAW 264.7	2.5, 5, 10 μmol/L	Inhibits osteoclast differentiation and bone resorption by targeting the TNF-α/NF-κB and SRC/PI3K/Akt signaling pathways	Yu et al. (2026)
*Achyranthes bidentata* Blume	Polysaccharide	Water extractio	ABW70-1	MC3T3-E1	50, 100 μg/mL	Stimulates osteogenic differentiation, upregulates gene expression of Osx, Ocn and Bsp	Zhang et al. (2019)
		Water extraction	ABW90-1	Primary osteoblasts	9.3, 93, 232.5 465 μmol/L	Promotes osteoblast differentiation	Wang et al. (2017)
		0.3 mol/L NaOH extraction	ABPB-4	MC3T3-E1	0.01, 0.02, 0.04 μmol/L	Promotes osteoblast proliferation, differentiation, and mineralization, and significantly upregulates the mRNA expression levels of osteogenic genes	Lin et al. (2021)
		—	ABP	BMMs	6.5, 8.5, 10 μmol/L	Inhibits of MAPK signaling pathway phosphorylation, downregulates of c-Fos-NFATc1 axis	Song D. et al. (2018)
	Steroid	Purified metabolite	β-Ecdysterone	MC3T3-E1	100, 150, 200 μmol/L	Induces bone regeneration through the BMP-2/Smad/Runx2/Osx pathway	Yan C. P. et al. (2022)

**TABLE 3:** Summary of *in silico* studies for the bioactive metabolites and mechanism prediction of representative anti-osteoporotic botanical drugs.

Source species	Metabolite/type of extract assessed	Prediction of bioactive metabolites	Signaling pathway prediction	Bibliography
*Epimedium brevicornu* Maxim	—	Epimedin A, Epimedin B, Epimedoside A, 4-Hydroxybenzaldehyde, Baohuoside VI	Targets: FAK1, FAK2 Molecular docking: EA-FAK1 (−13.012 kJ/mol), EA-FAK2 (−5.815 kJ/mol); EB-FAK1 (−14.0164 kJ/mol), EB-FAK2 (−6.4852 kJ/mol) Modulates the FAK signaling pathway	Wei Z. et al. (2025)
	—	Neochlorogenic acid, Sagittatoside B, Chlorogenic acid, Cryptochlorogenic acid, Baohuoside 1, Sagittatoside B, Cryptochlorogenic acid, Neochlorogenic acid, Chlorogenic acid, Icariin	High-score targets: INS, AKT1, IL6, TP53, TNF, VEGFA, MAPK3, EGFR, EGF, SRC, CASP3, MAPK1, STAT3, JUN, MYC, MAPK8, PTGS2, MMP9, IL10, and FOS FoxO signaling pathway, MAPK signaling pathway, TNF signaling pathway	Song et al. (2024)
	—	Quercetin, Epimedium B, Baohuoside I, anhydroicaritin-7-O-glucoside	Targets: AKT1, ABCB1, CYP19A1, EGFR Molecular docking: Quercetin-AKT1 (−5.53 kcal/mol), Quercetin-ABCB1 (−4.25 kcal/mol), Quercetin- CYP19A1 (−4.04 kcal/mol), Quercetin-EGFR (−6.68 kcal/mol); EB-AKT1 (−4.69 kcal/mol), EB-ABCB1 (−1.56 kcal/mol),EB- CYP19A1 (−0.35 kcal/mol), EB-EGFR (−2.87 kcal/mol); Baohuoside I -AKT1 (−5.17 kcal/mol), Baohuoside I -ABCB1 (−4.18 kcal/mol), Baohuoside I - CYP19A1 (−3.39 kcal/mol), Baohuoside I -EGFR (−6.65 kcal/mol); Anhydroicaritin-7-O-glucoside-AKT1 (−5.71 kcal/mol), Anhydroicaritin-7-O-glucoside-ABCB1 (−4.3 kcal/mol), Anhydroicaritin-7-O-glucoside - CYP19A1 (−5.77 kcal/mol), Anhydroicaritin-7-O-glucoside-EGFR (−5.83 kcal/mol) Influences Akt1 and PTGS2	Liang et al. (2025)
*Drynaria roosii* Nakaike	Water extraction	Kaempferol-3-O-rutinoside, Procyanidin B2, Prunin	Targets: AKT1, ESR1, ESR2, MMP9, SRC Molecular docking: Kaempferol-3-O-rutinoside-AKT1(-10.4 kcal/mol), Kaempferol-3-O-rutinoside-ESR1(-5.3 kcal/mol), Kaempferol-3-O-rutinoside-ESR2(-8.4 kcal/mol); Procyanidin B2-ESR1(-5.4 kcal/mol), Procyanidin B2-ESR2(-7.8 kcal/mol), Procyanidin B2-MMP9(-9.2 kcal/mol), Procyanidin B2- SRC(-8.1 kcal/mol); Prunin-AKT1(-9.2 kcal/mol), Prunin- ESR1(-7.8 kcal/mol), Prunin- ESR2 (−8.1 kcal/mol) Modulates the IR microenvironment through the ER/PI3K-EP300 signaling axis	Liu et al. (2025)
*Cullen corylifolium* (L.) Medik	Ethyl acetate partition fraction derived from the 75% ethanol extract	Bavachalcone, Isobavachalcone, Isobavachromene, BG, Bavachin, Genistein, Corylifol A, Neobavaisoflavone, Psoralidin	Targets: ERα, ERβ Bavachalcone-ERα/ERβ (−10.948/-10.276 kJ/mol); Isobavachalcone-ERα/ERβ (−11.4/-10.664 kJ/mol); Isobavachromene-ERα/ERβ (−7.269/−4.939 kJ/mol); BG-ERα/ERβ (−7.166/-7.614 kJ/mol); Bavachin-ERα/ERβ (−7.675/-8.53 kJ/mol); Genistein-ERα/ERβ (−9.998/-9.9 kJ/mol); Corylifol A-ERα/ERβ (−7.261/-9.751 kJ/mol); Neobavaisoflavone-ERα/ERβ (−10.991/-9.23 kJ/mol); Psoralidin-ERα/ERβ (−8.228/-10.474 kJ/mol) Activates of ER-Wnt-β-catenin signaling pathway	Cai et al. (2021)
*Eucommia ulmoides* Oliv	—	Cyrtominetin, Quercetin, Syringetin, Genistein, Ombuin, Kaempferol	Targets: RANKL; binding affinity: Total_Score >4.0 RANKL/RANK/OPG signaling axis	Zhang et al. (2025c)
*Achyranthes bidentata* Blume*-Dipsacus asper* Wall. ex DC.	—	Sitogluside, Arjunolic acid, Chondrillasterol, Stigmasterol, Spinasterol, Spinoside A, Cauloside A, Sylvestroside III, β-ecdysterone, Sitosterol, Oleanol, Baicalin	Targets: IGF1, MAPK1/8/14, AKT1, EGFR; all values ranged from −5.64 to −9.43 kcal/mol; MAPK signaling pathway (ERK/JNK/p38 cascade), PI3K-Akt signaling pathway, and osteoclast differentiation pathway	Li T. et al. (2023)
*Cistanche deserticola* Ma	—	Xanthoxylin N, Osthol, Diosmetin, (E)-2,3-bis (2-keto-7-methoxy-chromen-8-yl) acrolein, O-Acetylcolumbianetin	Targets: BCL2, CASP3, JUN, AKT1, PPARG Xanthoxylin N- BCL2/CASP3/JUN/AKT1/PPARG (-CIE = 22.6/27.27/30.18/31.79/32.02); Osthol- BCL2/CASP3/JUN/AKT1/PPARG (-CIE = 26.47/26.32/33.07/38.05/34.55); Diosmetin- BCL2/CASP3/JUN/AKT1/PPARG (-CIE = 26.39/34.22/34.59/44.33/40.70); (E)-2,3-bis (2-keto-7-methoxy-chromen-8-yl) acrolein- CASP3/JUN/AKT1/PPARG (-CIE = 43.43/42.93/56.19/47.64); O-Acetylcolumbianetin- BCL2/CASP3/JUN/AKT1/PPARG (-CIE = 31.16/31.47/35.47/40.55/36.71) PI3K-Akt, AGE-RAGE, TNF signaling pathway	Feng et al. (2025)
*Gynochthodes officinalis* (F.C.How) Razafim. and B.Bremer	—	Morindon, Ohioensin A, Physcion	Targets: IGF1R, INSR, ESR1, MMP9 Morindon-ESR1 (−9.0 kcal/mol); Morindon-MMP9 (−8.4 kcal/mol); Ohioensin A-IGF1R (−8.2 kcal/mol); Ohioensin A- INSR (−10.1 kcal/mol); Physcion-MMP9 (−8.5 kcal/mol); Physcion-ESR1(−8.3 kcal/mol) Ovarian steroidogenesis signaling pathway	Liu Z. W. et al. (2020)
*Cuscuta chinensis* Lam	—	Sesamin, NSC63551, Isofucosterol, Beta-sitosterol, Campest-5-en-3beta-ol, CLR, Isorhamnetin, Sophranol, Quercetin, Matrine, Kaempferol	Targets: IL6, NFATC1 Matrine-IL6 (−33.26 kJ/mol); Matrine-NFATC1 (−30.50 kJ/mol); Quercetin-IL6 (−27.95 kJ/mol); Kaempferol-NFATC1 (−26.07 kJ/mol) Oxidative stress and estrogen-related	Xu et al. (2026)
*Achyranthes bidentata* Blume	—	25R-inokosterone	Targets: PIK3CA, MTOR, TNF, MAPK3, CDK2, NTRK1 25R-inokosterone-PIK3CA/MTOR/TNF/MAPK3/CDK2/NTRK1 (−8.4/−8.3/−8.2/−7.9/−7.8/−6.9 kcal/mol) Regulates of PIK3CA/MTOR signaling pathway	Tan et al. (2025)

## 4 The main types of natural products for osteoporosis

As potential alternatives to anti-osteoporosis drugs, botanical drugs have increasingly attracted interest due to their diverse bioactive metabolites with anti-osteoporotic properties. Based on modern pharmacological research, flavonoids, saponins, and polysaccharides are the main metabolites in TCM botanical drugs used to treat osteoporosis. These metabolites primarily exert their therapeutic effects on osteoporosis by modulating the body’s hormone levels and calcium-phosphorus metabolism, exerting anti-inflammatory actions, inhibiting osteoclast activity, enhancing osteoblast proliferation, and improving bone microstructure (Wei F. et al, 2024; Zhu Z. et al, 2021; Yan C. P. et al, 2022).

### 4.1 Flavonoids

#### 4.1.1 *Epimedium brevicornu* Maxim

Flavonoids have been identified as the principal bioactive metabolites responsible for the anti-osteoporotic effects of *E*. *brevicornu*, with icariin (ICA) serving as the predominant bioactive metabolite (Yan et al., 2025). Using an ovariectomized (OVX) rat model and RANKL-induced RAW264.7 cells, Si et al. demonstrated that ICA suppresses Cullin 3 expression, thereby stabilizing Nrf2 activity and promoting its nuclear translocation, which subsequently activates the downstream Nrf2/HO-1 antioxidant pathway, reduces ROS generation, inhibits osteoclastogenesis, and alleviates osteoporosis (Si et al., 2024). Lin et al. revealed that ICA promotes osteogenic differentiation and inhibits adipogenic differentiation of ADSCs by suppressing the Hippo pathway, reducing YAP/TAZ phosphorylation levels and enhancing their transcriptional activity, thereby reducing the occurrence of osteoporosis (Lin et al., 2025). Collectively, these findings suggest that ICA bidirectionally regulates bone remodeling by suppressing resorption and enhancing formation. However, although ICA exhibits significant anti-osteoporotic activity, its poor water solubility and low permeability result in extremely low oral bioavailability (<1% in rats) (Ding et al., 2025). Therefore, high oral doses are typically required in preclinical models to achieve the pharmacological activity observed *in vitro* studies. Following oral administration, intestinal microbiota metabolizes ICA into icariside II and icaritin (ICT), which exhibit superior intestinal absorption compared to the parent compound ICA (Szabó et al., 2022; Hu et al., 2025). Ma et al. found that the inhibitory effect of icariside II on osteoclast differentiation is stronger than that of ICA at equivalent concentrations (0.01–1 µM) (Ma et al., 2022). Li et al. found that icariside II could also downregulate S100A16 and inhibit bone marrow adipogenesis induced by OVX (Li D. et al., 2024). These findings indicates that icariside II may prevent bone loss by inhibiting bone resorption and reducing adipogenic differentiation to promote bone formation. Huang et al. demonstrated that ICT inhibits osteoclastogenesis by suppressing the transcription factors NFATc1 and c-fos, alongside attenuating mitochondrial biogenesis essential for osteoclast differentiation. These actions collectively reduce bone resorption and ameliorate OVX-induced bone loss (Huang et al., 2023). Notably, few unmetabolized drug molecules are detected in plasma after oral administration of ICT, predominantly as glucuronide conjugates. The metabolic pathway is dominated by glucuronidation, accompanied by oxidation and demethylation (Gao and Zhang, 2022). It is worth noting that ICT can significantly inhibit drug metabolizing enzymes such as CYP1A2, 2C9, 3A4 and UGT1A1, 1A3, 1A7 (Gao and Zhang, 2022), suggesting that there is a potential risk of interaction between ICT and drugs metabolized by such enzymes.

In addition to icariin, the flavonoid metabolites of *E*. *brevicornu* include Epimedin A, B, and C (EA, EB, EC). Li et al. found that EA blocked RANKL-triggered signaling pathways, and negatively regulated osteoclastogenesis by inhibiting the TRAF6/PI3K/Akt/NF-κB to improve OP (Li J. et al, 2024). Zhang et al. demonstrated that EB alleviates weight loss and hyperglycemia in streptozotocin-induced diabetic osteoporosis (DOP) rats by modulating the OPG/RANKL pathway, while also enhancing BMD, improving bone microstructure, promoting bone formation, and suppressing bone resorption and inflammation (Zhang et al., 2025b). The study revealed that EC could restore the osteogenic differentiation of dexamethasone-injured MC3T3-E1 cells through activation of the PI3K/Akt/Runx2 signaling pathway, while zebrafish experiments indicated its efficacy against prednisolone-induced bone loss involves regulation of MAPK, PPAR, and estrogen signaling pathways (Xu et al., 2022; Zhou X. et al, 2024).

#### 4.1.2 *Drynaria roosii* Nakaike

*D. roosii* exerts multi-pathway and multi-factor regulatory effects on osteoblasts, osteoclasts, and MSCs, involving signaling pathways such as Wnt/β-catenin, MAPK, OPG/RANKL/RANK, Notch, BMP-Smads, and PI3K/Akt, as well as cytokines including TNF-α, IL-1, IL-6, estrogen and its receptors, and cathepsin K (Liu B. et al, 2024). The anti-osteoporotic flavonoid metabolites in *D*. *roosii* are represented by naringin, naringenin and neoeriocitrin. Naringin demonstrates dual regulatory properties against osteoporosis. Wang et al. demonstrated that naringin enhances the viability and osteogenic differentiation of bone marrow mesenchymal stem cells (BMSCs) by suppressing the JAK2/STAT3 signaling pathway, thereby mitigating bone loss and improving key parameters such as BMD and trabecular number, while also modulating bone metabolism markers (Wang et al., 2022). Additionally, studies have shown that naringin promotes nuclear translocation of ERα in both human and mouse osteoblasts, thereby upregulating ALP expression; in a mouse model of bone defect, it elevates local ERα/ALP levels, consequently enhancing bone mineralization, healing, and strength (Wu et al., 2021). Yang et al. revealed that naringin inhibits osteoclastogenesis by mediating the Wnt/β-catenin pathway, leading to an increased OPG/RANKL ratio (Yang et al., 2020). Naringenin could operate through antioxidant mechanisms. Cao et al. suggested Naringenin activates the Nrf2/HO-1 antioxidant axis to suppress ROS accumulation and mitochondrial dysfunction, thereby inhibiting NLRP3 inflammasome-mediated osteoblast pyroptosis and restoring osteogenic marker expression, ultimately ameliorating microgravity-induced bone loss (Cao S. et al, 2024). Moreover, both neoeriocitrin and naringin significantly downregulate mRNA expression of RANKL and SOST, while promoting the viability of osteocyte-like cells (Jin et al., 2022).

#### 4.1.3 *Cullen corylifolium* (L.) Medik

The flavonoids from *C*. *corylifolium* commonly used in the treatment of osteoporosis are neobavaisoflavone (NBIF) and corylin. Zhu et al. demonstrated that NBIF promotes osteoblast differentiation and exerts anti-osteoporotic effects by upregulating CRNDE, activating the Nrf2/HO-1 signaling pathway and enhancing antioxidant defense (Zhu Z. et al, 2021). Chen et al. isolated and identified NBIF as a metabolite with anti-osteoclastogenic activity. It could reduce osteoclast function by blocking RANKL-mediated TRAF6 and c-Src recruitment, reducing the activation of NF-κB, MAPKs, and Akt pathways, and preventing calcium oscillation and NFATc1 nuclear translocation (Chen et al., 2020). Corylin similarly exhibits pleiotropic bone-protective properties. Corylin attenuates postmenopausal osteoporosis by inhibiting osteoclast differentiation and suppressing RANKL-mediated NF-κB pathway activation (Li X. Q. et al, 2021). Yu et al. found that Corylin induced osteoblast differentiation by activating estrogen signaling pathway and Wnt/β-catenin signaling pathway. *In vitro* experiments showed that it could promote the activity and mineralization of osteoblasts and the expression of osteogenic markers such as Runx2, Osx, Col1, and ALP, regulate the OPG/RANKL ratio, and enhance mitochondrial function (Yu et al., 2020).

#### 4.1.4 Traditional medicine comparison of flavonoids

Although naringin is considered to be the main anti-osteoporotic metabolite in the traditional botanical drug *D. roosii*, it is also abundant in Mediterranean and North American plants, such as *Citrus × paradisi* Macfad., Rutaceae. and *Citrus × aurantium* L. These plants are widely used in food and show good anti-oxidative stress effects (Csuti et al., 2024; Stabrauskiene et al., 2025). Wang et al. elucidated that naringin promotes osteogenic differentiation under oxidative stress conditions through the activation of Wnt/β-catenin and PI3K/Akt signaling pathways, thereby facilitating bone formation and offering therapeutic potential for osteoporosis intervention (Wang H. et al, 2024). Furthermore, kaempferol and quercetin are ubiquitous bioactive flavonoid metabolites distributed across diverse ethnopharmacological traditions. These metabolites occur not only in TCM botanical drugs- including *E. brevicornu*, *C. chinensis*, and *E. ulmoides*- but also widely distributed in *Moringa oleifera* Lam. and *Emblica officinalis* Gaertn. Studies have shown that *M*. *oleifera* and *E*. *officinalis* have anti-osteoporotic effect (Hairi et al., 2025; Laldingliani et al., 2022). Mechanistically, *M. oleifera* by regulating the expression of Runx2 and BMP2 to regulate osteoblast differentiation, while reducing the RANKL/OPG ratio. It also activates the PI3K/Akt/Foxo1 pathway, reduces oxidative damage and promotes bone formation induction. In addition, it can also affect the expression of p38α/MAPK14 and RANKL/OPG ratio, reduce bone resorption and maintain the balance of bone remodeling (Hairi et al., 2025). *E. officinalis* showed anti-bone resorption activity by significantly altering the expression of Fas, NF-κB and IL-6, and by activating the programmed cell death of osteoclasts.

### 4.2 Saponins

#### 4.2.1 *Dipsacus asper* Wall. ex DC

Asperosaponin VI (AVI) is considered the main anti-osteoporotic metabolite of *D. asper*. Wei et al. discovered that AVI inhibits DNMT1/3a, reduces GPX4 promoter hypermethylation, restores GPX4 expression, ultimately alleviating osteoblastic ferroptosis and DOP-induced bone loss (Wei F. et al, 2024). Chen et al. demonstrated that AVI can promote the expression of osteogenic genes in BMSCs, inhibit RANKL expression, and reduce the differentiation of BMMs into osteoclasts. AVI can produce a synergistic effect with BS (BMP-2 fixed in 2-N, 6-O-sulfated chitosan), and significantly enhance osteogenesis, promote angiogenesis and inhibit osteoclast formation through activation of SMADs, TGF-β1, VEGFA and OPG/RANKL signaling pathways (Chen et al., 2021).

#### 4.2.2 *Panax ginseng* C. A. Mey

Research has demonstrated that various ginsenosides exhibit significant regulatory effects on bone metabolism. Currently, eleven ginsenosides have been identified to promote osteoblast activity, with Rb1 and Rc showing remarkable upregulation of key osteogenic markers including Runx2, ALP, COL-1, OCN, OPN, BMP-2, and β-catenin (Ko, 2024). Zhang et al. demonstrated that ginsenoside Rb1 enhances BMD and improves bone microstructure. *In vitro* studies revealed that Rb1 upregulates the aryl hydrocarbon receptor, promotes transcription of proline/arginine-rich end leucine-rich repeat protein, and subsequently inhibits the NF-κB pathway, thus enhancing osteoblast differentiation and upregulating osteogenic gene expression (Zhang et al., 2022). Meanwhile, *in vitro*, ginsenoside Rc stimulates osteogenic differentiation of MC3T3-E1 cells through Wnt/β-catenin pathway activation. *In vivo*, ginsenoside Rc treatment attenuated bone loss in OVX rats by upregulating bone formation-related genes and potentiating TGF-β/Smad signaling. Collectively, these observations indicate that ginsenoside Rc exerts osteoprotective effects through multiple mechanisms (Yang et al., 2022; Wang S. et al, 2024). Ginsenoside Rg1 enhances osteogenic differentiation and mineralization while upregulating osteogenic marker expression through GPER-mediated regulation of the PI3K/Akt pathway (Jiang et al., 2024). Hou et al. further revealed that Rg1 activates the Nrf2 signaling pathway to mitigate oxidative stress damage in BMMSCs, thereby restoring the osteogenic-adipogenic equilibrium compromised during aging (Hou et al., 2022).

Moreover, eight ginsenosides including Rb2 and Rg3 have been demonstrated to suppress osteoclastogenesis and osteoclast activity (Ko, 2024). Ma et al. revealed that ginsenoside Rb2 inhibits osteoclast formation and attenuates bone loss in orchidectomized mice through the NF-κB/MAPK signaling pathway (Ma et al., 2024). Similarly, Zhang et al. found that ginsenoside Rg3 can inhibit RANKL-induced osteoclast differentiation of RAW 264.7 cells by modulation of KPNA2 and the NF-κB signalling pathway (Zhang et al., 2025d).

#### 4.2.3 Traditional medicine comparison of saponins

Saponins represent one of the most extensively investigated classes of natural products. Among these, ginsenosides, the characteristic bioactive metabolites of *Panax* species, are abundantly present in *P*. *ginseng* and *Panax notoginseng* (Burkill) F.H. Chen, exhibiting diverse pharmacological properties. In the context of osteoporosis therapy, *P. notoginseng* has demonstrated significant anti-osteoporotic potential across multiple experimental models (Zhu et al., 2025). While sharing major ginsenosides (Rb1, Rd, Re, Rg1, Rg2, and Rh1) with *P. ginseng*, *P*. *notoginseng* uniquely produces notoginsenosides, such as R1, Rt, R2, and R3. Notably, notoginsenoside R1 ranks as the third most abundant saponin in *P. notoginseng* (Liu et al., 2020c). *In vitro* studies have demonstrated that notoginsenoside R1 alleviates oxidative stress-induced mitochondrial damage and restores osteoblast differentiation by inhibiting JNK pathway activation (Li X. et al, 2021). These findings highlight notoginsenoside R1 as a promising candidate for the treatment of osteoporosis.

### 4.3 Alkaloids

#### 4.3.1 *Botryodiscia tetrandra* (S.Moore) L. Lian and Wei Wang (syn. *Stephania tetrandra* S. Moore)

The primary bioactive alkaloid of *B. tetrandra* is tetrandrine (TET). Studies have demonstrated that TET promotes degradation of TRAIL protein, thereby inhibiting RANKL-induced osteoclast formation (Li et al., 2022). Furthermore, TET can suppress RANKL-induced osteoclast proliferation, reduce p-p65 levels, and modulate the expression of key factors such as RANKL, Ki67, PPAR-γ, ALP, and OPG. Additionally, TET suppresses the release of inflammatory cytokines and promotes macrophage transformation from the M1 to M2 phenotype, ultimately dampening osteoclast activity and preventing OVX-induced bone loss (Shi et al., 2022; Zhong et al., 2020). Similarly, fangchinoline, another bioactive alkaloid derived from *B. tetrandra*, suppresses RANKL-induced osteoclast formation and bone resorption by downregulating NFATc1 expression and transcriptional activity, thereby ameliorating OVX-induced bone loss *in vivo* (Zhou et al., 2020).

#### 4.3.2 *Coptis chinensis* Franch

The medicinal efficacy of *C. chinensis* is largely attributed to its alkaloids, with berberine being the principal bioactive metabolite responsible for its broad therapeutic benefits. In animal models, berberine has been demonstrated to inhibit ferroptosis *via* activation of the SLC7A11/GSH/GPX4 signaling pathway, thereby reducing osteoclast formation and alleviating bone loss induced by nonalcoholic fatty liver disease (Gu et al., 2024). Research indicates that berberine derivative compound 13 facilitates osteoblast differentiation through the Akt and PKC signaling pathways, as evidenced by molecular docking analysis; nevertheless, comprehensive mechanistic assessment and *in vivo* therapeutic efficacy warrant further investigation (Piao et al., 2025). Palmatine, another alkaloid metabolite, exhibits selective cytotoxicity toward osteoclasts over osteoblasts. Evidence suggests that palmatine promotes osteoclast apoptosis through the nitric oxide synthase system, consequently attenuating bone resorption and preventing osteoporosis (Ishikawa et al., 2016). Furthermore, Lee et al. discovered that coptisine downregulates RANKL expression while upregulating OPG expression. This alkaloid directly targets osteoclast precursors, suppressing RANKL-induced NF-κB p65 phosphorylation and NFATc1 nuclear translocation, ultimately reducing the survival rate of mature osteoclasts (Lee et al., 2012). Taken together, *C. chinensis* acts as a potential anti-osteoporotic drug candidate by synergistically suppressing osteoclast differentiation and function through its multi-component composition.

#### 4.3.3 *Sophora flavescens* Aiton

Matrine and its derivatives exhibit multi-target regulatory effects in osteoporosis treatment. Mechanistically, matrine suppresses osteoclastogenesis by inhibiting the activation of NF-κB, MAPK, and Akt signaling pathways, while downregulating the expression of osteoclast-specific markers including NFATc1, MMP-9, and TRAP (Chen et al., 2017). The matrine derivative M19 has emerged as a promising therapeutic candidate for postmenopausal osteoporosis. Zhou et al. demonstrated that M19 protects osteoblasts from DEX-induced apoptosis by inhibiting USP14-mediated p53 deubiquitination, thereby promoting p53 degradation (Zhou et al., 2019). Furthermore, oxymatrine suppresses RANKL-induced activation of sterol regulatory element-binding protein 2 and inhibits NFATc1 expression, consequently inhibiting ROS generation and reducing osteoclast formation and activity (Jiang et al., 2021). Additionally, oxymatrine ameliorates osteoporosis by modulating gut microbiota composition, reducing LPS release, and promoting osteoblast proliferation and mineralization through the miR-539-5p/OGN/Runx2 signaling pathway (Zhang Y. et al, 2025).

#### 4.3.4 Traditional medicine comparison of alkaloids

Berberine, a principal isoquinoline alkaloid found in *C*. *chinensis*, *Phellodendron chinense* C.K.Schneid., and *Berberis vulgaris* L., has shown significant anti-osteoporotic potential. Deng et al. identified berberine as a serum-absorbable metabolite of *P. chinensis* and demonstrated that *P. chinensis* monotherapy increased BMD and reduced NFATc1 levels in OVX mice. These protective effects were achieved through restoring the SLC7A11/GSH/GPX4 pathway and modulating the TFRC/ferritin pathway to maintain iron homeostasis (Deng et al., 2024). Consistently, Gu et al. demonstrated that berberine can inhibit ferroptosis by activating the SLC7A11/GSH/GPX4 signaling pathway, thereby reducing osteoclast formation (Gu et al., 2024). These findings underscore the potential of berberine as a novel therapeutic agent targeting ferroptosis for osteoporosis intervention.

### 4.4 Polyphenols

#### 4.4.1 *Reynoutria japonica* Houtt

*R. japonica* Houtt. is rich in resveratrol (RES), a natural phytoestrogen that functions as an ER agonist. Clinical studies have demonstrated that daily supplementation of 75 mg RES in postmenopausal women significantly reduces C-terminal telopeptide levels, improves BMD at the lumbar spine and femoral neck, enhances bone mineralization, increases osteocalcin levels, and effectively mitigates bone loss (Wong et al., 2020). Multiple studies have shown that RES inhibits RANKL-induced osteoclast differentiation by activating SIRT1 to inhibit NF-κB and MAPK pathways, while concurrently promoting osteoblast mineralization through upregulation of Wnt/β-catenin signaling (Shuid et al., 2025). These dual regulatory effects have been validated in animal models of postmenopausal, senile, glucocorticoid-induced, and disuse osteoporosis (Ahmad Hairi et al., 2023). Notably, as a potent antioxidant, RES can further alleviate high-altitude hypoxia-induced osteoporosis by inhibiting the ROS/HIF-1α axis and enhancing osteoblast formation (Yan C. et al, 2022).

#### 4.4.2 *Curcuma longa* L

A 6-month placebo-controlled clinical trial in 100 patients with spinal cord injury showed that daily administration of curcumin (CUR) at 110 mg/kg significantly increased femoral neck and total hip bone mineral density and reduced bone turnover marker levels (Hatefi et al., 2018). CUR shows a multi-target regulatory role in the treatment of osteoporosis, and its mechanism involves the regulation of intestinal microflora and bone metabolism signaling pathways. Li et al. demonstrated that curcumin treatment regulated intestinal microbial diversity in GIOP rats, altered the composition of intestinal microbial communities, modified serum metabolite profiles (including raffinose, ursolic acid, and spermidine), and reduced pro-inflammatory cytokine levels, ultimately exerting anti-osteoporotic effects through the “gut-bone axis” (Li S. et al, 2025). *In vitro* studies indicate that CUR protects osteoblasts from oxidative stress and promotes osteoblast formation by activating the GSK3β-Nrf2 signaling pathway, suggesting a novel therapeutic target for osteoporosis treatment (Li X. et al, 2020). Mohammad et al. reported that CUR further activates the SIRT3/FoxO3a signaling pathway to inhibit mitochondrial oxidative stress and apoptosis, promotes osteoblast proliferation and restores differentiation capacity, upregulates the expression of oxidative stress-related proteins Nrf2 and SIRT3 in bone tissue of type 2 diabetic osteoporotic rats, and improves trabecular microarchitecture (Mohammad et al., 2025).

#### 4.4.3 Traditional medicine comparison of polyphenols

Polyphenols are a class of metabolites widely distributed in botanical drugs with potent antioxidant activity. Saleh et al. found that *Cichorium intybus* L., a traditional botanical drug in Egypt, ameliorates GIOP by regulating RANK/RANKL/OPG and Nrf2/HO-1 signaling pathways (Saleh et al., 2024). This plant contains abundant polyphenols and RES, which are likely the key to its anti-osteoporotic effect through anti-oxidation and anti-inflammation.

### 4.5 Polysaccharides

#### 4.5.1 *Astragalus mongholicus* bunge

In recent years, growing attention has been drawn to the effects of Astragalus polysaccharides (APS) on osteoporosis among researchers. Liu et al. investigated the osteoprotective effects of APS via gut microbiota modulation. Their research demonstrated that APS significantly increased BMD and restored bone microarchitecture in OP rats, while reducing acid phosphatase 5 and pro-inflammatory cytokine levels. Notably, APS treatment markedly altered the gut microbiota composition, with c_Bacteroidia, p_Bacteroidetes, and g_Alloprevotella identified as potential biomarkers for APS-mediated OP improvement, suggesting a “gut-bone axis” mechanism of action. Furthermore, APS can increase the global CpG methylation level of colonic epithelial cells and increase the content of methyl donors such as SAM and betaine in cecum. It can restore intestinal function through epigenetic regulation, affect calcium homeostasis, osteoblast/osteoclast balance, and methylation of Wnt signaling pathway-related genes, promote calcium absorption, inhibit bone resorption, and improve bone microstructure (Liu et al., 2020d; Liu et al., 2021; Liu et al., 2020e).

#### 4.5.2 *Achyranthes bidentata* Blume

*Achyranthes bidentata* Blume has been traditionally employed in the treatment of osteoporosis, and investigations into its bioactive metabolites have predominantly centered on polysaccharides. *A*. *bidentata* polysaccharide (ABP) significantly inhibited the transcriptional activity of NFATc1 and reduced the phosphorylation level of MAPK signaling pathway by down-regulating RANKL-induced c-Fos and NFATc1 gene expression and related protein levels, thereby blocking osteoclast differentiation and weakening its bone resorption function (Song et al., 2018b). In OVX rat models, crude polysaccharide fractions (AB50, AB70, AB90) increased BMD and BMC, improved femoral biomechanical strength, and reduced bone turnover markers (Yan et al., 2019a; Zhang et al., 2019; Wang et al., 2017). Subsequent purification of these fractions has yielded distinct homogeneous saccharides. Yan et al. demonstrated that ABW50-1 significantly increased the relative fluorescence intensity of bone mass in a zebrafish model of GIOP, without observable adverse effects (Yan et al., 2019a). Zebrafish enable rapid screening of GIOP models, but their simpler skeletal physiology cannot fully recapitulate mammalian systems; thus, they serve only for preliminary exploration and require validation in mouse/rat models (Dietrich et al., 2021). Notably, these purified polysaccharides demonstrate markedly divergent *in vitro* potency. The fructan ABW90-1 promotes primary osteoblast proliferation and ALP activity at 9.3–465 μM (optimal concentration: 93 μM) (Wang et al., 2017), while ABW70-1 stimulates MC3T3-E1 cell osteogenic differentiation and mineralized nodule formation at 50–100 μg/mL (Zhang et al., 2019). ABPB-4 is a heteropolysaccharide, which can significantly promote the proliferation, differentiation and mineralization of MC3T3-E1 cells at the concentration of 0.01–0.04 μmol/L, and enhance the mRNA expression of osteogenic genes such as Runx2 and Osx, showing excellent osteogenic activity *in vitro*, and is expected to be used as an anti-osteoporotic drug (Lin et al., 2021). These studies have elucidated that *A*. *bidentata* polysaccharide can promote bone formation and effectively prevent and treat osteoporosis.

#### 4.5.3 Traditional medicine comparison of polysaccharides

Structurally diverse polysaccharides are ubiquitously distributed across various botanical drugs. Beyond those from *A. membranaceus* and *A. bidentata*, polysaccharides derived from other botanical drugs demonstrate significant anti-osteoporotic potential. Bu et al. demonstrated that *Dendrobium officinale* Kimura and Migo polysaccharide ameliorates OVX-induced bone loss through modulation of the gut-bone axis. This polysaccharide enriches SCFA-producing genera, restores the intestinal barrier integrity, suppresses inflammation, and activates the Wnt/β-catenin signaling pathway to promote bone formation (Bu et al., 2026). In addition, Feng et al. first identified that β-glucan-like polysaccharides isolated from *Pinellia ternata* (Thunb.) Makino had anti-osteoporotic activity. Both β-glucan-like polysaccharides (PTTP50-1-3 and PTTP50-1-4) could increase the ALP activity of MC3T3-E1 cells, and PTTP50-1-4 could also enhance the mineralization of MC3T3-E1 cells by up-regulating the expression of Runx2, Osx, Bmp2, Ocn, Opn and Bsp (Feng et al., 2026). These findings illustrate that plant polysaccharides exert anti-osteoporotic effects through multiple targets and pathways.

### 4.6 Other bioactive metabolites with anti-osteoporotic potential

In addition to the extensively investigated natural products mentioned above, researchers have also explored the anti-osteoporotic potential of various other metabolites, including terpenoids and coumarins, as well as animal-derived materials such as artificial tiger bone, deer antler, and turtle shell. These substances have been systematically studied and reported for their bone-protective effects through multiple mechanisms.

Triptolide, a diterpenoid metabolite derived from *Tripterygium wilfordii* Hook.f., has been demonstrated to effectively inhibit RANKL-induced NF-κB activation and suppress both RANKL- and tumor cell-mediated osteoclastogenesis (Wu D. et al, 2025). Mechanistic studies indicate that triptolide exerts its anti-osteoporotic effects by suppressing the PI3K/Akt/NFATc1 signaling cascade, downregulating Akt and NFATc1 phosphorylation, and upregulating murine double minute 2 expression, thereby inhibiting osteoclast differentiation (Cui et al., 2020).

Zhang et al. showed that isopsoralen, a furanocoumarin from *C. corylifolium*, promotes osteoblast proliferation and upregulates BMP2, Runx2, and Osx mRNA expression at an optimal concentration of 10 μg/mL (Zhang et al., 2023).

Velvet antler polysaccharides, polypeptides, and their mixtures can suppress bone resorption in high-turnover osteoporosis by downregulating the expression of ERK1, JNK, and MMP-9 genes and proteins via modulation of the MAPK and MMP-9 signaling pathways, thereby potentially alleviating retinoic acid-induced bone loss. Notably, velvet antler polypeptides exhibit more potent anti-resorptive effects than polysaccharides (Liu et al., 2023).

Furthermore, natural products can modulate the OP pathological process through multi-component and multi-target synergistic mechanisms. A single botanical drug of TCM inherently contains multiple bioactive metabolites that can simultaneously target different pathways, creating synergistic or complementary therapeutic effects. Using *C. corylifolium* as a paradigm, its flavonoid corylin inhibits osteoclastogenesis *via* NF-κB pathway blockade, while its coumarin isopsoralen concurrently enhances osteoblast activity through BMP2/Runx2/Osx signaling (Li X. Q. et al, 2021; Zhang et al., 2023). This multi-component, multi-pathway synergy provides natural products with unique advantages in improving bone microstructure.

Recently, naturally derived extracellular vesicle-like particles (EVLPs) have garnered extensive attention due to their unique advantages, serving as an important resource for osteoporosis treatment. Hwang et al. isolated Yam-derived exosome-like nanovesicles (YNVs) from *Dioscorea japonica* Thunb., which notably lack the traditional saponins diosgenin and dioscin. The YNVs significantly promote osteoblast proliferation, differentiation, and mineralization through activation of the BMP-2/p-p38 MAPK-dependent Runx2 signaling pathway, exhibiting superior osteogenic activity compared to diosgenin or dioscin. Oral administration of YNVs effectively alleviates bone loss in OVX mice (Hwang et al., 2023). Concurrently, Lu et al. purified EV-like particles (SP-EVLPs) from the traditional medicinal insect *Steleophaga plancyi* (Boleny), revealing that SP-EVLPs promote osteogenic differentiation by activating melatonin-induced autophagy, thereby significantly enhancing bone formation in OVX rat models (Lu et al., 2025). Collectively, these findings provide a critical foundation for the development of multi-targeted, low-toxicity oral bone-targeting therapeutics.

## 5 Conclusion and perspectives

As a complex metabolic bone disorder, OP involves an imbalance in the dynamic equilibrium between bone resorption and formation. In recent years, natural products have demonstrated promising therapeutic potential for OP treatment due to their unique chemical diversity and favorable safety profiles. This review summarizes the natural products from TCM with anti-osteoporotic activity. By categorizing representative botanical drugs and their metabolites, preclinical evidence from *in vitro* cell models and *in vivo* animal experiments is systematically integrated. The molecular mechanisms by which these metabolites exert preventive and therapeutic effects by modulating the balance between bone formation and resorption are further summarized. This review aims to provide a pharmacological basis and translational insights for developing natural products as alternative or adjuvant therapies for OP.

At present, the research on natural products from TCM against osteoporosis remains largely confined to the preclinical stage, focusing on molecular mechanism elucidation. However, several methodological limitations compromise the reliability and reproducibility of current findings. In animal studies, inadequate reporting of randomization and blinding, combined with small sample sizes and short treatment durations, increases the risk of experimental bias and limits generalizability. In cellular studies, most reports lack standardized descriptions of cell line authentication, hindering cross-laboratory reproducibility. Moreover, osteogenic responses observed under conventional plastic culture conditions may not reliably predict *in vivo* outcomes, and should therefore be regarded as preliminary mechanistic insights rather than therapeutic efficacy evidence. Moving forward, future research must adopt more rigorous experimental designs and encourage multi-team cross-validation of important findings to consolidate the evidence base in this field.

Equally concerning is that critical areas essential for clinical translation, such as pharmacokinetic characteristics, human-effective dosage ranges, and potential drug interactions, remain relatively understudied. This gap hinders the translation of experimental evidence into clinical applications. Many natural bioactive metabolites exhibit extremely low oral bioavailability due to intestinal enzymatic degradation and extensive first-pass metabolism, resulting in unpredictable systemic exposure. To address this, it is imperative to establish a standardized pharmacokinetic evaluation system, overcome absorption barriers through formulation optimization or structural modification, and systematically evaluate drug interactions, so as to promote the substantial transformation of anti-osteoporotic natural products from laboratory to clinical.

Furthermore, the clinical translation of natural products is severely constrained by batch differences and insufficient quality control standardization. For example, as the representative bioactive metabolite of *E*. *brevicornu*, ICA is still confronted with multiple limitations in its current quality standardization and regulatory evaluation system. The accuracy of its quantitative analysis is susceptible to interference from multiple factors, and the current quality control model, which relies on a single component, has inherent defects. These challenges extend beyond ICA and are systemic issues common to diverse natural products, including flavonoids, terpenoids, and alkaloids. A multi-link management and control system, including the construction of a comprehensive phytochemical fingerprint, the conduct of stability verification under various storage conditions, and the implementation of strict qualification audits for raw material traceability, should be established to ensure the consistency of clinical application effects (Noviana et al., 2022; Biotechnology Persa, 2025). It is necessary to further introduce modern analytical technologies and construct a full-process multi-component collaborative regulation model to improve the quality control system for natural products.

Currently, the development of bone-targeted delivery systems and the improvement of oral bioavailability are key strategies to promote the clinical translation of natural products for osteoporosis treatment. Studies have demonstrated that nanoformulation technologies can significantly enhance the water solubility and intestinal permeability of natural products (Zhang et al., 2024; Abdelnabi and Mohsin, 2025). Concurrently, functionalizing nanocarriers with bone-targeting ligands enables targeted drug accumulation in bone tissue, reduces systemic toxicity, and thereby achieves therapeutic efficacy at reduced doses (Dhiaa et al., 2025; Xi et al., 2022).

Collectively, natural products provide novel therapeutic perspectives and strategies for osteoporosis prevention and treatment, demonstrating substantial development potential.

## Glossary

OP: Osteoporosis
SERMs: Selective estrogen receptor modulators
TCM: Traditional Chinese medicine
SOST: Sclerostin
LRP5/6: low-density lipoprotein receptor-related protein 5/6
BMP: Bone morphogenetic protein
TGF-β: Transforming growth factor β
Runx2: Runt-related transcription factor 2
Osx: Osterix
ALP: Alkaline phosphatase
OCN: Osteocalcin
MSCs: Mesenchymal stem cells
RANKL: Receptor activator of nuclear factor-κB ligand
RANK: Receptor activator of nuclear factor-κB
OPG: Osteoprotegerin
M-CSF: Macrophage colony-stimulating factor
c-Fms: Receptor CSF1R
Grb2: Growth factor receptor-bound protein 2
ERK: Extracellular signal-regulated kinase
Akt: Protein kinase B
AR: Androgen receptor
TNC: Tenascin-C
ER: Estrogen receptor
ADSC: Adipose-derived stem cell
PR: Progesterone receptor
PN: Prednisolone
DOP: Diabetic osteoporosis
BMD: Bone mineral density
OVX: Ovariectomized
BMSCs: Bone marrow mesenchymal stem cells
BMMs: Bone marrow-derived macrophages
ICA: icariin
ICT: icaritin
EA, EB, EC: epimedin A, B, and C
NBIF: neobavaisoflavone
AVI: Asperosaponin VI
TET: Tetrandrine
RES: Resveratrol
CUR: Curcumin
APS: Astragalus polysaccharides
ABP: A. bidentata polysaccharide
EVLPs: Extracellular vesicle-like particles
YNVs: Yam-derived exosome-like nanovesicles
